# Global Disease Burden Attributable to Ambient Air Pollution: Disparities, Determinants, and Implications for Public Health

**DOI:** 10.34133/hds.0447

**Published:** 2026-07-03

**Authors:** Yixuan Jiang, Su Shi, Xia Meng, Haidong Kan

**Affiliations:** ^1^ School of Public Health, Key Lab of Public Health Safety of the Ministry of Education and NHC Key Lab of Health Technology Assessment, Fudan University, Shanghai 200032, China.; ^2^ Children’s Hospital of Fudan University, National Center for Children’s Health, Shanghai 201102, China.

## Abstract

**Background:** Ambient air pollution is a leading environmental risk factor for premature mortality worldwide. Characterizing the associated disease burden and spatiotemporal patterns is essential for informing global health policy. **Methods:** Using data from the Global Burden of Disease Study 2021, we assessed temporal trends and spatial heterogeneity in disease burden attributable to ambient fine particulate matter (PM_2.5_) and ozone (O_3_) during 1990–2020. We further examined the association between sociodemographic index (SDI) and disease burden, and conducted decomposition analysis to quantify the driving factors of temporal changes in disease burden. **Results:** From 1990 to 2020, the age-standardized death rates attributable to ambient PM_2.5_ and O_3_ both declined globally, but substantial regional heterogeneity was observed. High-SDI regions generally experienced declining burdens, whereas low- and middle-SDI regions showed persistent or increasing trends. A significant reversed U-shaped association between SDI and both PM_2.5_- and O_3_-attributable burden was observed, consistent with an environmental Kuznets-type pattern. Despite the decline in age-standardized rates, the absolute number of deaths attributable to ambient PM_2.5_ and O_3_ both increased, driven primarily by population aging, population growth, and increasing exposure levels, although reductions in baseline mortality rates partly offset these increases. **Conclusions:** Ambient air pollution continues to contribute to substantial and persistent disparities in global disease burden. Health-oriented and region-specific air pollution control strategies are needed to effectively mitigate these inequities and improve population health.

## Introduction

Air pollution is recognized as the leading environmental risk factor for morbidity and mortality worldwide. Robust epidemiological evidence has established causal links between major ambient air pollutants, including fine particulate matter (PM_2.5_), ozone (O_3_), and nitrogen dioxide, and a wide range of adverse health outcomes [1–3]. According to the Global Burden of Disease Study (GBD) 2023, approximately 8 million deaths could be attributable to air pollution exposure around the world [4].

Recent advances in satellite remote sensing, chemical transport modeling, and ground monitoring integration have improved the spatial and temporal coverage of population exposure estimates [4,5]. These progresses have, in turn, facilitated the expansion of epidemiological studies across diverse regions, yielding broader and more representative evidence linking air pollution to adverse health outcomes [4–6]. Based on these high-quality epidemiological studies, unified exposure–response modeling approaches such as the integrated exposure-response (IER) function, the meta-regression-Bayesian, regularized, trimmed (MR-BRT) tool, and the global exposure mortality model (GEMM) have been developed [5,7,8]. Together, these methodological innovations allow more comparable burden estimations across locations and years, making attributable disease burden data and risk assessments indispensable tools for evidence-based policymaking.

Globally, despite efforts to improve air quality in many countries, substantial spatial and temporal heterogeneity remains in both pollution levels and the associated disease burden [4,9,10]. High-income regions have generally achieved substantial declines in ambient pollutant concentrations, whereas many low- and middle-income regions continue to face high exposure levels and disproportionately heavy attributable disease burden [4,9]. These disparities reflect differences in socioeconomic development, emission control capacity, demographic transitions, and baseline health conditions [11]. Understanding this heterogeneity is particularly important for health-oriented identification of priority control areas [12], an approach that focuses on maximizing health benefits by targeting populations and regions with the greatest potential burden reduction.

Therefore, the present study characterizes global variation in air pollution-attributable disease burden and quantifies key drivers of its temporal change. Based on annual estimates of exposure, sociodemographic index (SDI), population data, mortality data, and attributable burden from the GBD Study from 1990 to 2020, we assess (a) the association between SDI and disease burden across 21 GBD regions, and (b) the contributions of population growth, population aging, baseline mortality, and pollutant exposure to changes in burden over time.

## Methods

### Overview

The analyses in this study were based on estimates from the GBD Study 2021. The overall design, data inputs, and standardized analytical methodologies of GBD Study have been described in detail elsewhere [5]. Briefly, GBD 2021 provides annual, age- and sex-specific estimates of disease burden attributable to major air pollutants for 204 countries and territories around the world. For this study, we extracted annual mean concentrations of ambient PM_2.5_ and O_3_, SDI values, and age-standardized death rates attributable to these pollutants for 21 GBD regions as well as the global level from 1990 to 2020. In addition, we also obtained global population number, age structure, and baseline mortality rates for 1990 and 2020 to support the decomposition analyses of temporal changes in burden. Pollutant data were obtained from the State of Global Air platform (https://www.stateofglobalair.org/), while other data were obtained from the Global Health Data Exchange GBD Results Tool (https://gbd2021.healthdata.org/gbd-results/). Because the GBD database contains only aggregated, de-identified information, ethical approval and informed consent were not required.

### Associations between SDI and air pollution-attributable disease burden

To examine the relationship between socioeconomic development and air pollution-attributable disease burden, we assessed the association between SDI and age-standardized death rates across the 21 GBD regions from 1990 to 2020 using an environmental Kuznets curve (EKC) method [13]. The EKC assumes an inverted U-shaped quadratic relationship, in which disease burden initially increases and subsequently declines with socioeconomic development. Accordingly, the association was modeled as a second-order polynomial function:

$$Y=\beta_{0}+\beta_{1}\mathrm{SDI}+\beta_{2}{\mathrm{SDI}}^{2}+\varepsilon$$ (1)

where Y represents the age-standardized death rate; $\beta_{0}$ is the intercept; $\beta_{1}$ and $\beta_{2}$ denote the coefficients of the linear and quadratic SDI terms, respectively; and ε is the random error term. Evidence supporting an EKC pattern was defined as statistically significant coefficients for both the linear and quadratic terms (*P* < 0.05) with $\beta_{1}\;$> 0 and $\beta_{2}\;$< 0, indicating an inverted U-shaped relationship.

### Decomposition of drivers of temporal change in disease burden

We quantified the contributions of 4 major drivers, including population growth, population aging, changes in baseline mortality, and changes in air pollution concentrations, to temporal changes in attributable disease burden. A decomposition approach was applied to partition the net change in attributable deaths into contributions from each driver [14,15]. Starting from a baseline scenario in which all factors were fixed at their 1990 levels, we introduced each factor sequentially by replacing its 1990 value with the corresponding 2020 value, while previously updated factors remained at 2020 levels and the remaining factors stayed at 1990 levels. The contribution of each factor was calculated as the change in attributable burden between 2 consecutive scenarios. Because the results may depend on the order of factor introduction, we further applied a Shapley value-based averaging method, in which all possible replacement orders were considered and averaged. This approach provides an additive and order-independent estimates of the contribution of each driver to the total change in disease burden.

## Results

### Global trends and spatial patterns of ambient air pollution and disease burden

Over the past decades, marked changes have been observed in global ambient air pollution levels and the corresponding disease burden (Table 1). Globally, the population-weighted PM_2.5_ concentrations declined from 36.5 μg/m^3^ in 1990 to 32.5 μg/m^3^ in 2020. In contrast, ground-level O_3_ concentrations have increased from 43.9 to 49.8 μg/m^3^. There was substantial geographic heterogeneity. In many high-income regions, particularly North America and Europe, ambient PM_2.5_ and O_3_ concentrations have declined considerably, while South and East Asia, North Africa, and Middle East continue to report the highest levels of ambient PM_2.5_ and O_3_ worldwide.

**Table 1.** Ambient PM_2.5_ and O_3_ concentrations and attributable age-standardized death rates in 21 GBD regions during 1990 and 2020. Data for age-standardized death rates are shown as rates (95% uncertainty intervals) per 100,000.

	Concentrations (μg/m^3^)		Age-standardized death rate (per 100,000)	
	1990	2020	1990	2020
PM_2.5_				
Global	36.5	32.5	66.9 (48.2, 86.3)	55.4 (40.9, 68.6)
Andean Latin America	66.0	27.1	78.2 (37.4, 128.7)	36.8 (22.7, 53.2)
Australasia	6.8	6.9	16.1 (0.9, 43.2)	6.3 (3.4, 9.5)
Caribbean	13.0	13.3	48.9 (19.6, 91.7)	41.1 (21.5, 62.5)
Central Asia	26.8	27.4	87.2 (34.6, 158.7)	94.3 (66.7, 124.1)
Central Europe	29.8	16.5	118.2 (60.9, 179.0)	47.0 (36.7, 57.6)
Central Latin America	25.8	19.7	67.4 (36.9, 99.8)	32.8 (23.8, 42.4)
Central Sub-Saharan Africa	33.0	32.2	33.3 (17.9, 54.2)	35.5 (20.3, 53.0)
East Asia	42.4	33.2	73.9 (35.1, 129.3)	96.0 (66.3, 118.5)
Eastern Europe	30.3	13.2	137.9 (72.6, 205.6)	44.2 (26.2, 68.0)
Eastern Sub-Saharan Africa	20.5	23.1	19.4 (12.9, 28.7)	18.7 (11.7, 27.8)
High-income Asia Pacific	15.3	14.0	32.3 (9.7, 62.9)	12.7 (7.1, 19.5)
High-income North America	15.3	7.6	40.2 (17.2, 67.7)	9.0 (4.9, 13.6)
North Africa and Middle East	33.7	35.5	119.2 (87.8, 149.1)	99.5 (79.8, 118.6)
Oceania	12.8	11.7	30.6 (8.8, 73.6)	37.0 (11.6, 88.5)
South Asia	53.1	54.3	46.0 (24.9, 72.9)	76.7 (48.4, 103.2)
Southeast Asia	35.8	22.3	43.6 (19.6, 76.7)	57.9 (37.0, 78.5)
Southern Latin America	21.5	16.9	55.7 (28.3, 91.7)	28.4 (16.4, 42.0)
Southern Sub-Saharan Africa	22.0	24.6	52.7 (33.7, 72.3)	63.1 (43.7, 82.4)
Tropical Latin America	27.1	13.8	45.8 (16.3, 85.4)	22.5 (12.8, 34.0)
Western Europe	21.5	10.3	57.4 (30.0, 90.4)	11.6 (8.0, 15.8)
Western Sub-Saharan Africa	51.3	50.4	58.5 (33.8, 86.5)	57.0 (30.8, 85.8)
O_3_				
Global	43.9	49.8	6.8 (1.5, 11.9)	5.9 (1.3, 10.3)
Andean Latin America	29.0	36.2	0.4 (0.1, 0.7)	0.6 (0.1, 1.1)
Australasia	28.2	29.5	0.3 (0.1, 0.7)	0.3 (0.1, 0.6)
Caribbean	36.8	34.2	0.8 (0.1, 1.5)	0.6 (0.1, 1.1)
Central Asia	51.9	49.4	4.6 (0.9, 8.1)	2.3 (0.4, 4.1)
Central Europe	46.9	41.2	3.1 (0.7, 5.5)	1.1 (0.2, 1.9)
Central Latin America	47.9	38.3	4.3 (1.0, 7.1)	1.4 (0.3, 2.5)
Central Sub-Saharan Africa	42.7	47.0	4.1 (0.7, 7.8)	4.5 (0.7, 8.9)
East Asia	46.6	48.5	21.9 (4.6, 38.7)	7.4 (1.6, 13.1)
Eastern Europe	44.7	36.5	3.3 (0.7, 5.9)	0.5 (0.1, 0.9)
Eastern Sub-Saharan Africa	32.8	42.0	1.4 (0.3, 2.7)	2.2 (0.4, 4.1)
High-income Asia Pacific	40.9	49.5	0.9 (0.2, 1.6)	0.7 (0.2, 1.3)
High-income North America	48.0	42.5	2.8 (0.6, 4.9)	2.1 (0.5, 3.7)
North Africa and Middle East	51.3	53.6	4.6 (0.9, 8.2)	3.5 (0.7, 6.1)
Oceania	19.9	17.6	0.4 (0.1, 1.0)	0.3 (0.1, 0.5)
South Asia	49.1	66.7	13.7 (2.7, 24.9)	22.5 (4.8, 38.6)
Southeast Asia	29.5	37.2	1.2 (0.2, 2.5)	2.6 (0.5, 4.6)
Southern Latin America	34.5	35.4	0.9 (0.2, 1.7)	0.9 (0.2, 1.7)
Southern Sub-Saharan Africa	33.6	40.9	1.2 (0.2, 2.2)	2.2 (0.4, 3.9)
Tropical Latin America	33.0	40.6	1.2 (0.3, 2.3)	1.8 (0.4, 3.2)
Western Europe	42.6	42.8	1.7 (0.4, 3.0)	1.3 (0.3, 2.3)
Western Sub-Saharan Africa	41.2	50.6	2.0 (0.4, 3.6)	2.6 (0.6, 4.6)

PM_2.5_, fine particulate matter; O_3_, ozone; GBD, Global Burden of Disease Study

The age-standardized death rates attributable to ambient PM_2.5_ fell from 66.9 per 100,000 in 1990 to 55.4 per 100,000 in 2020, and those due to O_3_ declined from 6.8 to 5.9 per 100,000 over the same period. There was also substantial spatial heterogeneity in the disease burden. For PM_2.5_, age-standardized death rates are consistently higher in Africa and Asia, while lower levels are observed in North America, Western Europe, and Australasia. Over time, high-SDI regions generally experienced stable or declining PM_2.5_-attributable burden, whereas many low- and middle-SDI regions show slower decreases or continued increases, with the most pronounced rises observed in South Asia, Southeast Asia, and East Asia. For O_3_, age-standardized death rates are highest in South Asia. The largest increases during 1990–2020 are in low- and low-middle SDI regions, particularly in Southeast Asia and sub-Saharan Africa. Conversely, higher-SDI regions experience only small increases or even declines.

### Associations between SDI and air pollution-attributable disease burden

**Fig. 1.**
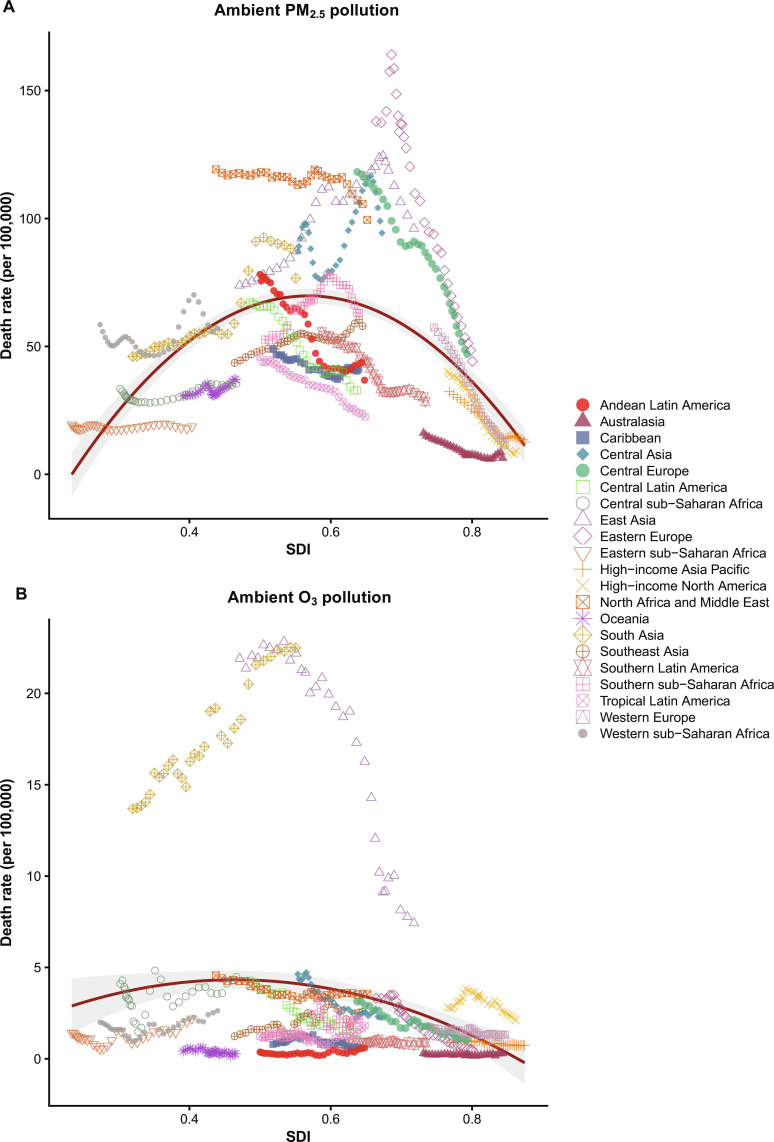
The relationships between SDI and age-standardized death rates attributable to ambient PM_2.5_ and O_3_ in different GBD regions during 1990–2020 [data source: Global Burden of Disease Study 2021 (https://gbd2021.healthdata.org/gbd-results/)]. Panel (A) shows the relationship for ambient PM_2.5_, and panel (B) shows the relationship for O_3_. The relationship was modeled by including both linear and quadratic terms of SDI. SDI, sociodemographic index; PM_2.5_, fine particulate matter; O_3_, ozone; GBD, Global Burden of Disease Study.

Nonlinear associations were observed between SDI and disease burden attributable to ambient air pollution (Fig. 1). For PM_2.5_, there is a reversed U-shaped curve between SDI and age-standardized death rates, with the greatest burden occurring in regions with middle and low-middle SDI. Specifically, the burden increased with SDI at lower development levels, peaked at an SDI of approximately 0.57, and subsequently declined as SDI increased further. Both the linear and quadratic terms were statistically significant (*P* < 0.05), supporting a robust nonlinear association consistent with the EKC hypothesis. The association between SDI and O_3_-attributable burden was weaker, characterized by a shallow inverted U-shaped curve with a turning point at an SDI of around 0.46, although both the linear and quadratic terms remained statistically significant (*P* < 0.05).

### Drivers of temporal changes in air pollution-attributable disease burden

**Fig. 2.**
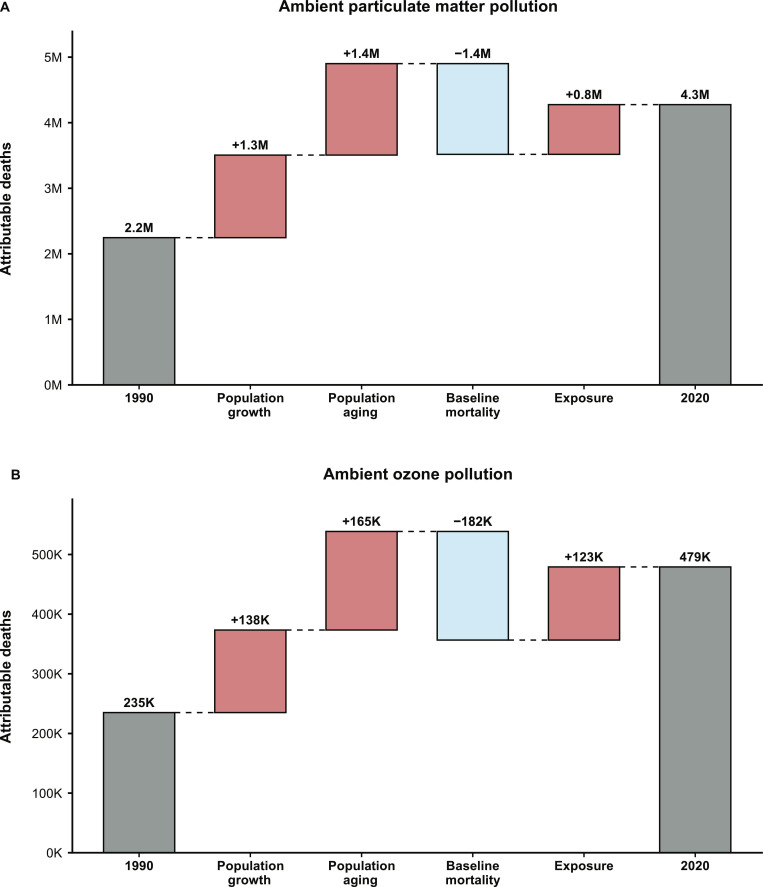
Decomposition of changes in PM_2.5_- and O_3_-attributable deaths from 1990 to 2020 [data source: Global Burden of Disease Study 2021 (https://gbd2021.healthdata.org/gbd-results/)]. Panel (A) presents the decomposition for ambient PM_2.5_-attributable deaths, and panel (B) presents the decomposition for O_3_-attributable deaths. PM_2.5_, fine particulate matter; O_3_, ozone.

Over the past 3 decades, the absolute number of deaths attributable to PM_2.5_ and O_3_ has increased globally (Fig. 2). Decomposition analysis indicated that demographic factors contributed the largest positive changes, while reduction in baseline mortality acted as the main mitigating factor. For PM_2.5_, attributable deaths increased from 2.2 million in 1990 to 4.3 million in 2020. Population aging and population growth were the dominant drivers of this increase, contributing approximately 1.4 million (68.8%) and 1.3 million (62.1%) additional deaths, respectively. Increases in PM_2.5_ exposure contributed an additional 0.8 million (37.4%) deaths, while decrease in baseline mortality offset part of this increase, reducing the burden by approximately 1.4 million (-68.2%) deaths. For O_3_, attributable deaths increased from 235 thousand in 1990 to 479 thousand in 2020. Population aging (165 thousand deaths; 67.7%) and population growth (138 thousand deaths; 56.7%) were the dominant contributors to this increase, followed by rising O_3_ exposure (123 thousand deaths; 50.2%). Declines in baseline mortality offset part of this increase, reducing deaths by approximately 182 thousand (−74.6%).

## Discussion

Based on data extracted from GBD 2021, this study examines the spatiotemporal trends in disease burden attributable to ambient air pollution exposure globally during 1990–2020. Regional disparities were evident, with high-SDI regions generally experiencing declining burden, while low- and middle-SDI regions continue to show persistent or increasing patterns. Over time, the number of attributable deaths continued to rise, driven primarily by increasing exposure levels, population aging, and population growth, while declines in baseline mortality have partly offset these increases.

The pronounced reversed U-shaped association between SDI and PM_2.5_-attributable mortality offers important insights into the development trap faced by middle-SDI regions. In low-SDI settings, limited industrial activity and the dominance of communicable diseases in the disease spectrum can obscure the relative impact of air pollution on overall mortality rates. At higher SDI levels, advanced environmental regulations, cleaner technologies, and improved baseline health jointly reduce both exposure and vulnerability. However, in middle-SDI regions where the burden peaks, rapid industrialization, urbanization, and energy consumption coincide with an epidemiological transition toward noncommunicable diseases that are highly sensitive to air pollution, creating a perfect storm for elevated health impacts. The formal statistical confirmation of this EKC-type relationship underscores the need for health-oriented environmental policies that are tailored to different stages of socioeconomic development and that integrate both exposure reduction and population vulnerability. In contrast, the SDI-O_3_ relationship was weaker, even though both the linear and quadratic SDI terms used to test the EKC hypothesis were statistically significant. This is partly due to the fact that ambient O_3_ has a much higher background level and substantial natural sources [16], which makes it less responsive to socioeconomic development compared with PM_2.5_. Still, the reversed U-shaped pattern suggests that similar mechanisms may emerge as O_3_ exposure and precursor emissions continue to rise in low- and middle-SDI countries. In addition, as O_3_ formation is strongly influenced by climate conditions, global warming may amplify these disparities [17].

Beyond socioeconomic drivers and epidemiological transitions, heterogeneity in policy timing and regulatory capacity further contribute to the regional disparities. Many high-SDI countries typically introduced stringent air-quality controls earlier and benefited from strong enforcement capacity and long-term structural transitions in energy and transportation systems [11]. Historical pollution crises, such as the 1952 London smog, played an important role in accelerating these policy shifts and prompting landmark regulations including the UK Clean Air Act 1956, U.S. Clean Air Act, and the European Union Ambient Air Quality Directives [18,19]. Still, it is worth noting that increasing wildfire smoke episodes have partly offset these long-term improvements in the United States in recent years [20]. In contrast, many low- and middle-SDI regions implemented air-quality interventions only recently [21,22]. For example, China launched a series of ambitious air-quality improvement policies, including the Air Pollution Prevention and Control Action Plan (2013–2017), the Three-Year Action Plan to Win the Blue Sky (2018–2020), and the Continuous Improvement of Air Quality Action Plan (since 2023) [23]. It is estimated that the annual average population-weighted PM_2.5_ concentrations were reduced by 19.8 and 10.9 μg/m^3^ during 2013–2017 and 2018–2020, respectively [23]. India’s Graded Response Action Plan, implemented in 2017 to mitigate severe pollution episodes in Delhi and the surrounding regions, has also been associated with reductions in the frequency of extremely high PM_2.5_ days in some areas [24]. These regional differences underscore not only the unequal progress in global air-quality improvement but also the urgent need for stronger international cooperation, particularly as many regions with the greatest pollution challenges face the weakest regulatory and technological capacities.

Decomposition analyses highlight demographic dynamics as the dominant contributors to the rising global air pollution-attributable deaths. Population aging alone accounted for approximately two-thirds of the increase for both PM_2.5_ and O_3_, reflecting growing vulnerability as age-related health conditions become more prevalent. Population growth further amplified the absolute burden in regions with rapidly expanding populations. Notably, reductions in baseline mortality offset a substantial portion of the pollution-related burden, demonstrating that advances in healthcare and chronic disease management remain critical for mitigating the health impacts of air pollution. Besides, increases in O_3_ exposure accounted for a relatively larger share of the rise in O_3_-attributable disease burden than the share contributed by increasing PM_2.5_ exposure to the PM_2.5_-attributable burden. This distinction indicates that future changes in O_3_ levels may pose a growing threat to global health.

Although this study benefits from the standardized and globally comparable framework of the GBD methodology, several important limitations remain and should be considered when interpreting the findings.

First, substantial uncertainties remain in the assessment of air pollution exposure, particularly in many countries from Africa. On the one hand, high-resolution and accurate exposure data remain scarce in these regions, and the lack of fixed-site monitoring makes it difficult to validate global model estimates [9]. On the other hand, the predictive accuracy of different models, or of the same model across different regions, varies and can affect study results. In addition, exposure assessment in GBD is based on country-level population-weighted average concentrations, which may mask substantial within-country spatial heterogeneity. This may lead to exposure misclassification, particularly in large countries with strong urban–rural gradients or marked regional variation in air pollution levels. Therefore, more advanced approaches with higher spatial resolution, broader coverage, and improved ability to capture individual-level exposures are still needed.

Second, the lack of comprehensive and high-quality population health data remains a major challenge for disease burden assessment, particularly in many low-SDI countries. In these settings, incomplete health surveillance systems, limited diagnostic capacity, and underreported morbidity and mortality make it difficult to obtain accurate health outcome data [9]. These limitations further hinder the estimation of disease burden. Therefore, strengthening health information systems and improving the availability and quality of population health data are therefore essential steps in the future.

Third, the complexity of air pollution exposure has not been adequately addressed. The GBD framework relies primarily on total PM_2.5_ mass and does not differentiate the heterogeneous toxicity of specific chemical constituents or sources, despite substantial evidence demonstrating heterogeneous health impacts of different chemical constituents and sources [25,26]. In addition, the potential interactions among multiple air pollutants are also overlooked, although real-world exposures typically involve complex pollutant mixtures rather than isolated pollutants [27]. Future studies should aim to refine the characterization of particulate matter, quantify the burden of more pollutants, and develop approaches that capture the health impacts of multi-pollutant exposures.

Fourth, as emerging studies support associations between air pollution and wide-spectrum outcomes [28], expanding burden assessments to cover a broader range of diseases (e.g., O_3_-related cardiovascular impacts) will be essential [29,30]. In addition, future work should better integrate both long-term and short-term health effects of air pollution, as current burden estimates are primarily derived from cohort-based evidence focusing on chronic exposures and may not fully capture acute mortality impacts on different temporal scales [31]. Such efforts help provide a more comprehensive picture of the overall impacts of air pollution and help guide effective public health interventions.

Last, current burden assessments rely on generalized exposure–response functions derived mainly from high-income countries [4,5]. However, exposure levels and toxicity, population characteristics, and healthcare conditions vary substantially across regions, making such extrapolation uncertain. In addition, current burden estimation frameworks assess individual diseases separately, which may lead to overlap of attributable burdens across related disease categories when risks are driven by shared pathophysiological pathways. Addressing these challenges will require the development of region-specific exposure–response relationships, as well as methodological advances in concentration–response modeling, including more flexible nonlinear functions and integrative approaches that better account for correlated outcomes.

## Conclusion

This study provides an overview of the global disease burden attributable to ambient air pollution, highlighting substantial spatial, temporal, and sociodemographic disparities. Although disease burden has declined in some regions, particularly in high-SDI countries with improved air quality, many low- and middle-SDI countries continue experiencing considerable burden. Overall, population aging, population growth, and worsening air quality have jointly driven increases in air pollution-related deaths, whereas declines in baseline mortality have partly offset these increases. Despite recent methodological and data advances within the GBD framework, important limitations remain in current exposure and disease burden assessment frameworks. Future research integrating high-quality health surveillance data and exposure data, expanded epidemiological evidence, and advanced methods for evidence synthesis are warranted for more precise evaluation of air pollution-related disease burden and for informing evidence-based policy decisions globally.

## Ethical Approval

There was a waiver of ethical approval for this analysis because GBD data are anonymous and publicly accessible.

## Data Availability

To download GBD data used in the analyses, please visit the Global Health Data Exchange GBD 2021 website (https://vizhub.healthdata.org/gbd-results/).
